# A Few Other Charities

**Published:** 1891-12-19

**Authors:** 


### A FEW OTHER CHARITIES.

**British Home for Incurables**, Clapham. (Office, 73, Cheapside, E.C.)—Helpless! Hopeless!! Homeless!!! These pathetic words form the motto of the British Home for Incurables, at Clapham, which national charity, in addition to providing for the nursing and comfort, but *not expensive luxury*, of the suffering inmates of the home, gives no less than £6,000 a-year in pensions to those incurably afflicted in all parts of the United Kingdom. Secretary, Mr. Robert G. Salmond.

**Christian Literature Society for India**, 7, Adam Street, Strand, W.C.—This Society seeks to provide suitable literature for the twelve millions of people who have been taught to read in Government Mission Schools in India.

**City of London Truss Society**, 35, Finsbury Square.—For the relief of the ruptured poor throughout the kingdom. At the present time about 10,000 of both sexes and all ages are treated annually, while over 500,000 patients have been relieved since its formation eighty-five years ago. Secretary, Mr. John Whittington.

**Cripples' Home and Industrial School**, 17a, Marylebone Road, N.W. This charity, which was established in 1851, is for females only. Hon. Secretaries, Miss Wellesley and Mr. E. Bannister.

**Home for Little Boys**, Farningham, Swanley, Kent.—An institution whose object is to receive all little lads, whether orphan or not, who, being homeless and destitute, are likely to fall into crime from lack of a friendly hand to save them. About 500 boys are provided for by this Institution in their homes at Farningham and Swanley, where they are trained from an early age till they become sufficiently expert at one of the many trades taught here to enter on life's duties fully equipped for their part in it. We commend this Institution to the notice of those who have little lads of their own. Secretary, Mr. B. Clarke, Bank Buildings, Ludgate Circus, E.C.

**Invalid Children's Aid Association**, 18, Buckingham Street, Strand, W.C.—To aid in every way that it is possible the invalid and crippled children of the poor in London is the aim of this Association, and up to the present, so far as its resources have permitted, it has endeavoured to carry out this worthy aim. Its *modus operandi* is, first, to find each child a friend, who shall give it, so far as circumstances permit, unstinted personal service. This being done, skilled nursing, medical advice, surgical appliances, admission into hospitals and homes, are often required, and here again the Society steps in, and as far as possible makes the necessary provision. With such worthy aims, we think liberal assistance should be forthcoming this Christmas. Subscriptions should be sent to the Hon. Secretaries.

**Irish Distressed Ladies' Fund**, 17, North Audley Street, W.—The Committee appeal for funds to enable them to help those ladies of Ireland who depend for their support on the proceeds of Irish property, but who, owing to the rent difficulty and causes beyond their own control, have been reduced to absolute poverty. Relief is given independently of any question of politics or religion; employment is found, when possible, for those able to work; pecuniary help is given to the aged and infirm; and the education of children is, in cases, paid for. Secretary, Lieut.-General W. M. Lees.

**London Orphan Asylum**, Watford.—This institution, for the maintenance and education of fatherless children of both sexes, is dependent for nine-tenths of its income upon voluntary subscriptions, and we trust that it may meet with the cordial recognition it deserves. 112 children have been admitted during this year, and an expenditure of £900 has been incurred in perfecting the sanitary arrangements at the asylum, so that the society stands in the most urgent need of help. Secretary, Mr. James Rogers, 21, Great St. Helen's, E.C.

**Metropolitan Drinking Fountain and Cattle Trough Association.**—The relief afforded to both human beings and dumb animals by this Society is incalculable, as it is the only one which provides free supplies of water for man and beasts in the streets of London. It is entirely dependent on voluntary contributions. Secretary, Mr. M. W. Milton, 70, Victoria Street, Westminster, S.W.

**Mrs. Hilton's Crèche**, 12, 14, and 16, Stepney Causeway, E.—For nearly twenty years this most useful institution has been steadily working its way amongst the poor classes of the East-end, and supplying a long-felt need. Receiving and tending some eighty to a hundred children of all ages daily, it at once benefits both the child and the hard-working and poverty-stricken mother, who (unable, through poverty, illness, or the necessities of her daily toil, to tend her child during the day), can leave it in safety in this friendly shelter. The children, whose ages vary from three weeks to five years, are washed, clothed, fed, and tended for twelve hours each day for the nominal charge of twopence a-day. Mrs. Hilton's Crèche, whilst it relieves the parent of the burden of its care, lays the foundation of better health in the child by providing good food and careful nursing. Contributions should be sent to Mrs. Hilton, 12, Stepney Causeway, E.

**National Health Society.**—The basis of the whole system of instruction which this most useful Society comprises, may be said to lie in the proverb that "Prevention is better than Cure." It goes straight to the root of the matter, as its aim is to diffuse by all possible means sanitary knowledge of every kind, comprising home nursing, rearing of infants, prevention of the spread of infectious diseases, and by no means least, important lectures on sanitary reform in food and cookery. The Society gives courses of lectures on the various subjects which affect health, not only in London and its vicinity, but even so far afield as Glasgow and Aberdeen. We wish this useful Society all success in its work. Secretary, Miss Lankester, 53, Berners Street, W.C.

**Zenana, Bible, and Medical Mission,** 2, Adelphi Terrace, W.C.—This society has a threefold object in its work, addressing itself to the spiritual, mental, and bodily evils under which the unhappy women of India have groaned for centuries. It is worthy of help from all. Especially should it appeal to women, for their sisters in India can only be reached by women; their sufferings only alleviated by women; and only women can enter their homes and personally influence them in mind and body. The great need of the mission at the present time is for friends of the work to subscribe to the purchase of a site for a hospital in connection with the medical mission recently opened at Patna. The house at present occupied is quite inadequate to the needs of the work, and is, moreover, insanitary. No more suitable house being available, there is no alternative but to build, a capital site being obtainable in the market place. It is estimated that £1,000, beyond what the society now has in hand, would enable them to acquire the site and build the hospital premises at once. So energetic is the society that we hope the generosity of English men and women will enable them to found this much-needed institution. Hon. Finance Secretary, Mr. W. T. Paton.

---

The following are deserving institutions to the work of which we would also call the attention of the charitable :—

### General Hospitals.

French Hospital, 172, Shaftesbury Avenue, W.C.—Secretary, M. F. Sorel.

London Temperance Hospital, Hampstead Road, N.W.—Secretary, Mr. E. W. Taylor; Matron, Miss S. E. Orme.

### Consumption.

Royal National for Consumption, Ventnor.—Secretary, Mr. E. Morgan, 34, Craven Street, W.C.; Lady Secretary, Miss Moore.

### Children.

Alexandra Hospital for Children, 18, Queen Square, Bloomsbury, W.C.—Matron, Miss L. E. Moore.

Belgrave Hospital for Children, 79 Gloucester Street, Pimlico.—Hon. Secretaries, the Rev. J. Storrs and Mr. Percy Gates; Lady Superintendent, Miss Munro.

Cheyne Hospital, 46, Cheyne Walk, S.W.—Honorary Secretary, Mr. Reginald Blunt; Matron, Miss Elam.

Home for Incurable Children, 2, Maida Vale, W.—Matron, Miss Coleman.

### Fever.

London Fever Hospital, Liverpool Road, N.—Secretary, Major W. Christie; Matron, Miss H. F. Ingall.

### Skin.

London Skin Hospital, 47, Cranbourne Street, Leicester Square, W.C.—Secretary, Mr. H. A. Gifford.

### Dental.

Dental, 40, Leicester Square, W.C.—Secretary, Mr. F. J. Pink.

National Dental, 149, Great Portland-street.—Secretary, Mr. Arthur G. Klugh.

### Ophthalmic.

Central London, 238, Gray's Inn Road.—Secretary, Mr. William Abrams; Matron, Mrs. Hugall.

Royal Westminster, King William-street, W.C.—Secretary, Mr. T. Beattie-Campbell; Matron, Miss Hordern.

Western, 153, Marylebone Road.—Secretary Mr. G. E. Manton.

### Lying-in.

British Lying-in, Endell Street, W.C.—Secretary, Mr. Fitzroy Gardner; Matron, Mrs. E. Freeman.

City of London Lying-in, City Road, E.C.—Secretary, Mr. R. A. Owthwaite; Matron, Miss S. E. Allen.

General Lying-in, York Road, Lambeth, S.E.—Secretary, Miss Annie Whyte; Matron, Miss Atkinson.

### A Few Other Charities.

Field Lane Refuges and Ragged Schools, Vine Street, Clerkenwell Road, E.C.—Secretary, Mr. Peregrine Platt.

Ragged School Union.—Secretary, Mr. John Kirk, Exeter Hall, W.C.

Religious Tract Society, 56, Paternoster Row, E.C.—Secretaries, Rev. L. B. White and Rev. S. Green.

Royal Albert Orphan Asylum, Bagshot, Surrey.—Secretary, Mr. R. Wetherby, 62 King William Street, E.C.

Royal Society for the Prevention of Cruelty to Animals, 105, Jermyn Street, S.W.—Secretary, Mr. John Colam.

Thames Church Mission, 31, New Bridge Street.—Secretary, Rev. H. Bloomer.

